# Identification of Transcriptional Modules and Key Genes in Chickens Infected with* Salmonella enterica* Serovar Pullorum Using Integrated Coexpression Analyses

**DOI:** 10.1155/2017/8347085

**Published:** 2017-04-26

**Authors:** Bao-Hong Liu, Jian-Ping Cai

**Affiliations:** ^1^State Key Laboratory of Veterinary Etiological Biology, Key Laboratory of Veterinary Parasitology of Gansu Province, Lanzhou Veterinary Research Institute, Chinese Academy of Agricultural Sciences, Lanzhou, Gansu, China; ^2^Jiangsu Co-Innovation Center for Prevention and Control of Important Animal Infectious Diseases and Zoonoses, Yangzhou, China

## Abstract

*Salmonella enterica *Pullorum is one of the leading causes of mortality in poultry. Understanding the molecular response in chickens in response to the infection by* S. enterica* is important in revealing the mechanisms of pathogenesis and disease progress. There have been studies on identifying genes associated with* Salmonella *infection by differential expression analysis, but the relationships among regulated genes have not been investigated. In this study, we employed weighted gene coexpression network analysis (WGCNA) and differential coexpression analysis (DCEA) to identify coexpression modules by exploring microarray data derived from chicken splenic tissues in response to the* S. enterica *infection. A total of 19 modules from 13,538 genes were associated with the Jak-STAT signaling pathway, the extracellular matrix, cytoskeleton organization, the regulation of the actin cytoskeleton, G-protein coupled receptor activity, Toll-like receptor signaling pathways, and immune system processes; among them, 14 differentially coexpressed modules (DCMs) and 2,856 differentially coexpressed genes (DCGs) were identified. The global expression of module genes between infected and uninfected chickens showed slight differences but considerable changes for global coexpression. Furthermore, DCGs were consistently linked to the hubs of the modules. These results will help prioritize candidate genes for future studies of* Salmonella* infection.

## 1. Introduction

**Figure 1:**
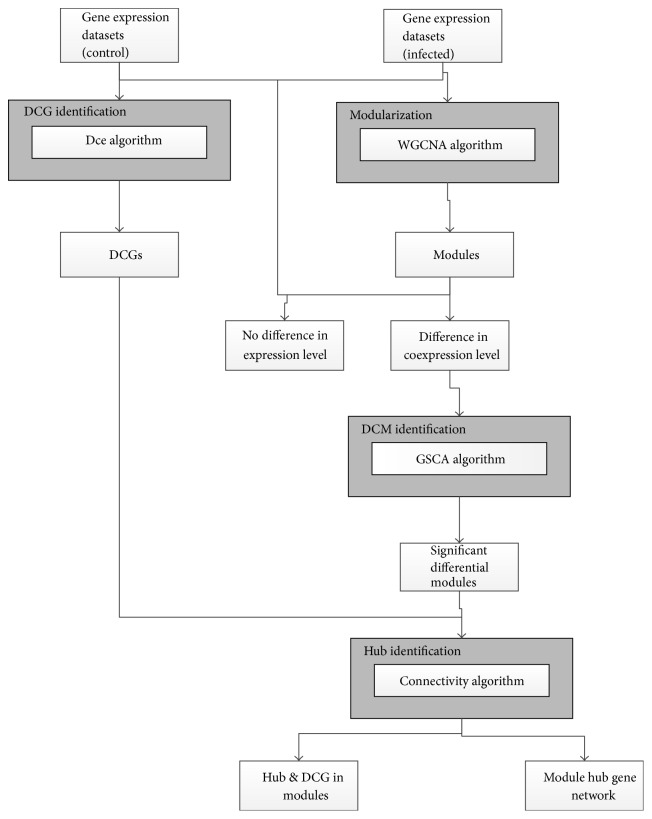
Workflow of the comprehensive gene coexpression network analysis.

Chickens are an important component in the global agricultural economy by serving as one of the primary sources of proteins for humans. However, the poultry industry has been consistently threatened by various diseases, including those caused by viral, bacterial, and parasitic infections.* Salmonella enterica *serovar Pullorum (*S*. Pullorum) is one of the most important pathogens of poultry causing severe systemic disease [1, 2]. To prevent and control *S*. Pullorum in chickens, the host responses against this pathogen have been studied for decades. Although significant advances have been made, especially in the identification of molecules and genes involved in the host immune response [3, 4] and mucosal inflammation [5, 6], as well as their differential expression during infection [7–10], the precise pathways regulating immunity to* Salmonella* infection using a systems biology approach have not been investigated. Although gene differential expression analysis (DEA) provides important information, such as identification of genes that are expressed at different times during infection, which inform our understanding of pathogenesis, identifying gene interactions using a systems biology approach greatly enhances our knowledge at the mechanistic and regulatory levels. A large amount of information regarding gene interactions is available in microarray datasets and by applying network approaches the gap between individual genes and systems can be bridged [11–13]. The modularity in biological systems allows for both the study of independent components and identification of gene relationships within modules. Modern approaches, such as weighted gene coexpression network analysis (WGCNA) [14], can identify modules with expression levels that are highly correlated across samples and have been used to identify new candidate regulatory molecules and networks in* Salmonella*-infected pigs [15]. Differentially coexpressed modules (DCMs) can also be identified [16]. The holistic changes in modules would be reflected in transcriptional and coexpression changes for individual genes. In general, gene expression levels change during disease or infection, but some have reported that seemingly nonsignificant DEGs may also play a key role in a disease because their interactions with other genes change considerably [17]. These genes can be identified via differential coexpression analysis (DCEA), which can mine individual genes using a holistic approach [17–19]. Hence, combining the WGCNA and DCEA methods can identify interacting modules and differentially coexpressed genes (DCG) during infection, compared with controls. Here, we mined the molecular network relationships of the differential coexpression modules and genes using microarray data from spleens of *S*. Pullorum*-*infected and uninfected chickens using WGCNA and DCEA (Figure 1). The results complement traditional DEA and add to our understanding of the regulatory mechanisms that occur during* Salmonella *infection.

## 2. Materials and Methods

### 2.1. Microarray Data Harvesting and Processing

A comprehensive transcriptomics dataset derived from microarray analysis of spleens from chickens challenged with 10^8^ CFU of* Salmonella enterica *serovar Pullorum or mock-challenged with the same volume of distilled water (controls) was obtained from the Gene Expression Omnibus (GEO) database (https://www.ncbi.nlm.nih.gov/geo/) (accession number: GSE59663). The dataset was generated with the Agilent oligo microarray chips containing 43,663 probe sets. In this study, we first streamlined the dataset by excluding 14,920 probe sets that were either unmappable to any gene IDs or mapped to multiple gene IDs. In the case of multiple probe sets mapped to one identical gene, the probe set, which is most often associated with the highest expression level, was maintained to ensure that only one probe set was left to investigate one gene. If more than one probe set was left after the above steps, their intensities were averaged. Finally, a one-to-one match between 13,538 probe sets and 13,538 genes was achieved.

Three biological replicates (chips) for each time point were available in the challenged group for these datasets. However, at each time point in the control group, only one chip was used to hybridize with the equally mixed mRNA sample containing the three control samples. We averaged the replicates for each time point, except at day 21, with the two replicates included and forming the dataset for the challenged group with 10 samples; this dataset was equivalent to the dataset of the control group. The dataset was quantile normalized by the function of normalizeQuantiles in R package limma [20].

### 2.2. Construction of Weighted Gene Coexpression Network and Identification of Modules

Weighted gene coexpression network analysis (WGCNA) was used to detect coexpression modules from the dataset of challenged samples [14, 21]. The R function of blockwise modules was implemented with the following parameters: power = 12, minModuleSize = 100, and networkType = “signed.” Microarray data were processed as described below.

The pairwise Pearson's correlation coefficients were calculated for all the genes in the challenged groups, followed by the construction of an adjacency matrix using the power function:

(1) $$\begin{array}{c} \alpha_{ij}={\left(\;0.5+0.5\times \mathrm{cor}\left(\;x_{i},x_{j}\;\right)\;\right)}^{\beta }, \end{array}$$

where *x*_*i*_ and *x*_*j*_ were the *i*th and* j*th gene expression traits, respectively, which formed a signed weighted correlation network; and *β* used default value (i.e., *β* = 12). The topological overlap measure (TOM) was calculated as follows:

(2) $$\begin{array}{c} TOM_{ij}=\frac{\sum_{u\neq i,j}\alpha_{iu}\alpha_{uj}+\alpha_{ij}}{\min\left(k.{total}_{i},k.{total}_{j}\right)+1-\alpha_{ij}}, \end{array}$$

where *k*.total is the sum of connection strengths for a gene with the other network genes. *u* is the other network genes.

Afterwards, 1-TOM was calculated as a biological important measure for network interconnectedness. Genes with highly similar coexpression relationships were grouped together by performing hierarchical clustering on the topological overlap. Subsequently, genes were hierarchically clustered using 1-TOM as the distance measure and modules were determined by choosing a height cutoff of 0.995 for the resulting dendrogram. Highly similar modules were identified by clustering and merged together using a dynamic tree-cutting algorithm [14]. Eigengene refers to the first principal component for a given module and could be calculated to draw a module trajectory curve [14].

### 2.3. Identification of Differentially Coexpressed Modules

Differentially coexpressed modules (DCMs) were identified using gene-set coexpression analysis (GSCA) that adopted the length-normalized Euclidean distance to measure the coexpression difference for the pairwise correlations between infected and control groups [16].

(3) $$\begin{array}{c} D_{m}=\sqrt{\frac{1}{P_{m}}\overset{P_{m}}{\underset{p=1}{\sum }}{\left(r_{p}^{c}-r_{p}^{i}\right)}^{2}}, \end{array}$$

where *P*_*m*_ was the number of gene pairs from the pairwise correlation for all the module genes.  *r*_*p*_^*c*^ and *r*_*p*_^*i*^ were the correlation coefficients for a gene pair in the control and infected groups, respectively.

The null distribution for distance was constructed by permuting samples across conditions for 10,000 times to yield gene-set specific *p* values. Modules with *p* value < 0.01 were considered as significantly differentially coexpressed.

### 2.4. Identification of Differentially Coexpressed Genes

The differential coexpression analysis (DCEA) was implemented by using R package DCGL, which is a useful tool to identify differentially coexpressed genes (DCGs) and differentially coexpressed links (DCLs) [17–19]. The R function DCe was applied and then the *p* values were adjusted for a false discovery rate (FDR) using the Benjamini-Hochberg method to reduce a large amount of false positive results [22]. The genes with FDR < 0.001 were selected as DCGs.

### 2.5. Gene Ontology (GO) and Pathway Enrichment for Coexpression Modules

GO enrichment and KEGG pathway analyses for network modules were performed using Database for Annotation, Visualization, and Integrated Discovery (DAVID, v6.7) program using all chickens genes as the background [23, 24]. The modified Fisher's exact test with an adjustment for multiple tests by Benjamini-Hochberg method was used to identify significantly enriched terms for module genes [22].

### 2.6. Network Visualization

The complex network bioinformatics software Cytoscape (v3.1.1) was used to visualize the pairwise relationships between genes [25].

## 3. Results

### 3.1. Weighted Gene Coexpression Network Analysis

**Figure 2:**
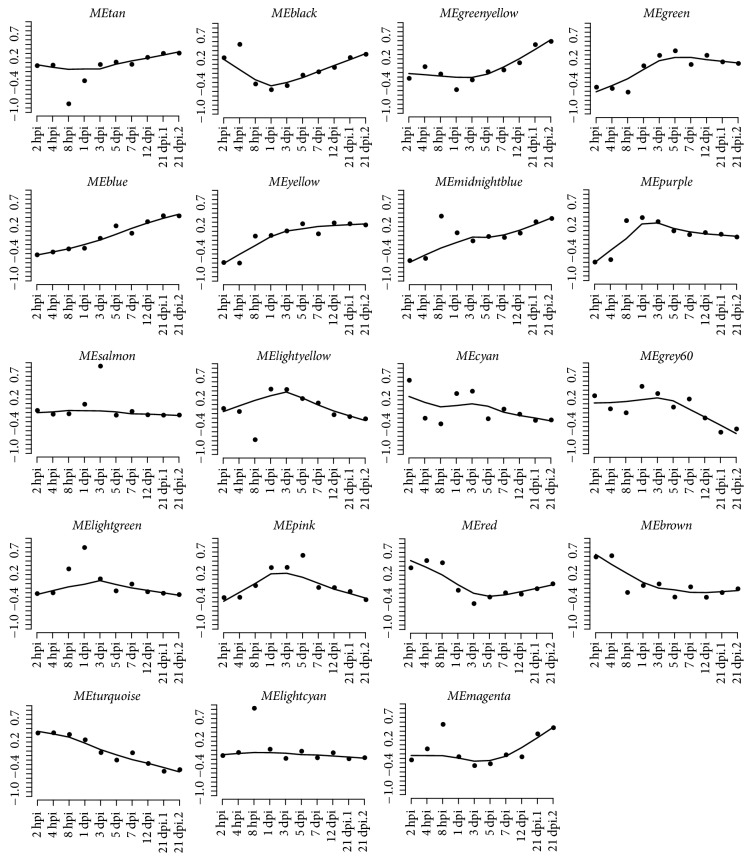
Module expression patterns.

Using blockwiseModules R function (*β* = 12), a total of 19 modules ranging from 100 to 3,000 genes were recovered for the 13,538 distinct genes in the* S*. Pullorum-infected group (Table 1). Each module was assigned a unique color, including gray color for the 373 unassigned genes. Genes in the same module shared the same or similar expression patterns that were catalogued by the trajectory curves (Figure 2).

Subsequent analysis using DAVID identified biological features in modules that were potentially associated with the infection by *S*. Pullorum (Figure 6 and Table 1), such as the Jak-STAT signaling pathway (module lightgreen) [26], the extracellular matrix (ECM) (module grey60) [27], cytoskeleton organization (module green), regulation of the actin cytoskeleton (module blue) [28], G-protein coupled receptor activity (module magenta), Toll-like receptor signaling pathways (module purple), and immune system processes (module blue). ECM genes and cell adhesion genes are significantly enriched in the module grey60 and cyan (FDR = 5.10*e* − 4 and 2.75*e* − 3), respectively (Table 1). The grey60 and cyan modules also displayed significant similarity in expression patterns (eigengenes' correlation = 0.76; *p* = 0.01). These observations were in congruent with those reported earlier by others on the crucial role of host cell ECM proteins and bacterial outer membrane structures in the adhesion and invasion of* Salmonella* [27].

### 3.2. Module Stability

**Figure 3:**
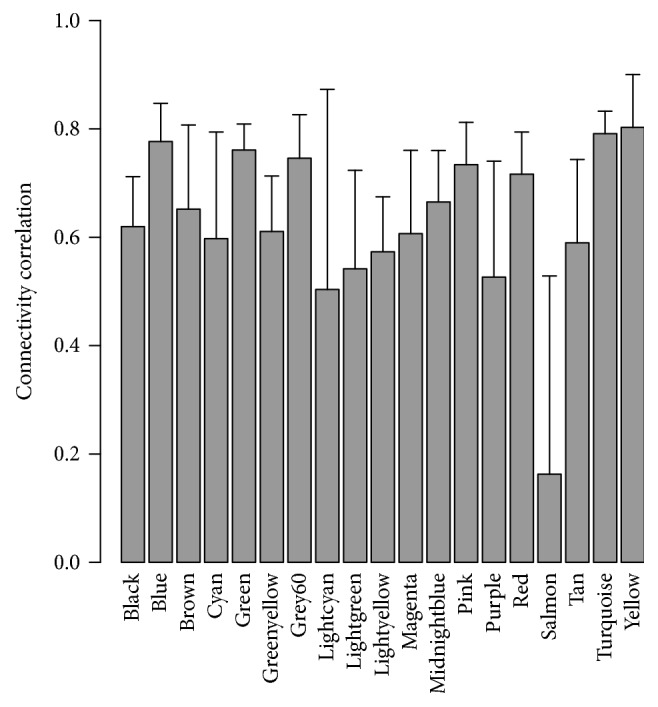
Correlation of intramodule connectivity for each module after 100 samplings.

To test the reproducibility of the identified modules, we performed a sampling test, in which we randomly selected half of the samples to calculate the new intramodule connectivity. The sampling was repeated 100 times and then the module stability was expressed as the correlation of intramodule connectivity between the original and sampled ones [29]. Most modules displayed good stability; module salmon was the least stable (Figure 3).

### 3.3. Module Preservation Analysis

We investigated whether the* S*. Pullorum-infected module was preserved in the corresponding controls by testing whether the infection-associated coexpression network can be replicated in the control groups. The preservation scores for all the modules were listed in Table 1, in which *Z* summary scores <2, between 2 and 10, and >10 indicate no evidence, weak-to-moderate evidence, and strong evidence for module preservation, respectively. Preservation analysis provided strong evidence to support the conservation of modules turquoise, brown, blue, yellow, green, red, pink, and purple, which all contained considerably large numbers of genes, but no evidence to support the preservation of modules lightcyan, lightgreen, magenta, and black associated with the membranes, the Jak-STAT signaling pathway, G-protein coupled receptor activity, and synapses, respectively (Table 1).

### 3.4. Module Gene Expression and Coexpression Comparison

**Figure 4:**
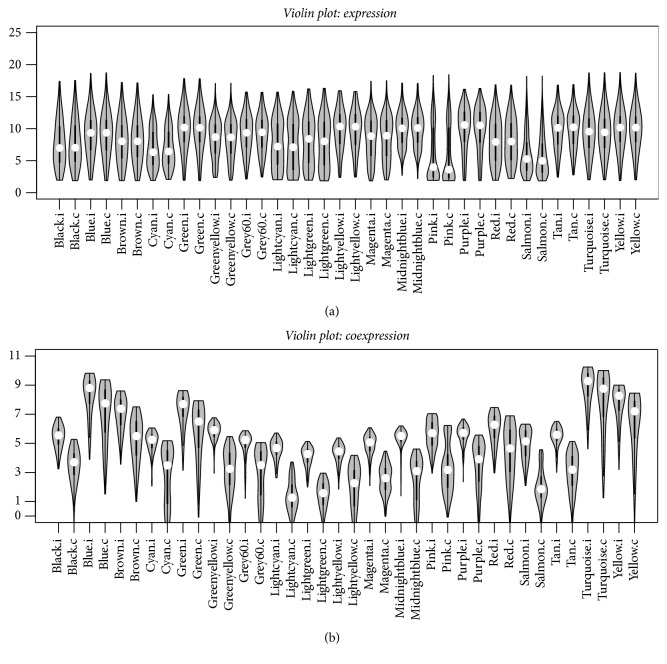
(a) Violin plot showing the gene expression differences between modules in the infected and control groups. (b) Violin plot showing the gene coexpression connectivity differences between modules in the infected and control groups.   .i represents the module in the infected group, and  .c represents the module in the control group.

We compared the module genes' expression and coexpression level between the infected and control groups. The violin plot in Figure 4(a) showed that the gene expression for modules in the infection versus control groups is not significantly different, and the distribution for the expression intensities is similar. Subsequently, we compared the gene coexpression level by calculating the gene connectivity for each module. The module turquoise exhibits the largest connectivities since it includes the largest number of genes (2,998 genes). Modules blue (2,581 genes), yellow (1,122 genes), brown (1,349 genes), and green (1,056 genes), which include a considerable number of genes, display the next highest connectivities. In addition, the coexpression levels are different between modules in the two conditions. The coexpressions are strengthened in the infected state (Figure 4(b)).

### 3.5. Identification of Differentially Coexpressed Modules

Gene-set coexpression analysis (GSCA) revealed that 14 of the 19 modules were significantly differentially coexpressed (*p* < 0.01 by bootstrap sampling test) (Table 2). Among them, modules black (*z* = 1.97), magenta (*z* = 1.82), salmon (*z* = 2.14), and lightcyan (*z* = 0.52) were significantly differentially coexpressed. These observations were in agreement with the module preservation analysis, in which significantly differentially coexpressed modules (DCM) were only weakly preserved in the control group.

### 3.6. Identification of Differentially Coexpressed Genes

A total of 2,856 differentially coexpressed genes (DCG) were selected with a false discovery rate (FDR) of less than 0.001 using the DCe method in the DCGL package. And a total of 284,213 differentially coexpressed links (DCLs) were same signed, 82,619 were differently signed, and 272,491 were switched links.

Furthermore, we mapped the DCGs for each module and found that the DCMs enrich the DCGs. For example, a total of 152 DCGs appeared in the module magenta with *Z* summary of 1.82 (*p* = 0), 231 DCGs in module black with *Z* summary of 1.97 (*p* = 0), and 147 DCGs in module salmon with *Z* summary of 2.14 (*p* = 0). In network biology, a hub gene is a good representative of a module. We identified the hub genes for all of the modules. Table 2 gives the gene names which are not only hub genes but also DCGs in each module.

## 4. Discussion

We constructed a gene network for the *S*. Pullorum-infected chickens using weighted gene coexpression network analysis (WGCNA) from the data of time-series microarray. This module detection strategy utilizes the biological variability inherent in the prospective cohort study to reveal the modular organization and function of transcriptional systems. The time series expression profiles allow the study of the transcriptional regulation of these gene coexpression networks during infection. A network-based analysis provides a systems-level understanding of the relationships between members of a network by focusing on genome-wide gene modules rather than individual genes [30]. Differential expression analysis (DEA) aims to identify genes that are expressed significantly higher or lower in one group compared with another. By contrast, WGCNA is not biased toward genes with significant changes in expression. Moreover, the dimensionality of microarray data in the present study was reduced from 13,538 genes to 19 modules, which significantly increased the ability to identify concordant changes in the expression of multiple genes.

**Figure 5:**
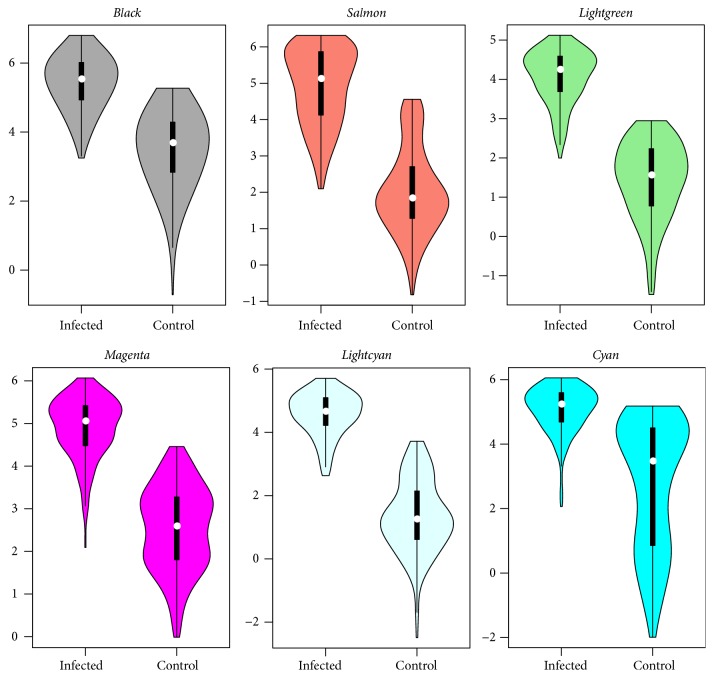
Violin plot of gene coexpression connectivity for significantly differentially coexpressed modules.

The module expression analysis showed that module salmon was the least abundant but exhibited the largest variation in gene expression causing the instability in module construction. Gene expression was the most stable within module midnightblue (Figure 4(a)). The expression distribution for module genes in different conditions (infected versus control) was the same. The coexpression level was further compared. The coexpression comparison showed significant changes for different conditions. We investigated the key modules and genes resulting in the differences. The GSCA analysis identified ten significantly differentially coexpressed modules (DCMs), which are in accordance with the module preservation results that are significant in coexpression differences with little evidence for preservation. Regulatory relationships among genes can be parsed as the pairwise correlations between gene expression levels, so the changes in coexpression patterns between two conditions may indicate dysfunctional regulatory systems in disease [31]. Module lightgreen associated with the Jak-STAT signaling pathway and lightcyan associated with membrane anchoring functions were the two weakest preserved modules (Table 1 and Figure 5). Thus, these modules may be associated with* S*. Pullorum infection in chickens.

Furthermore, we investigated the driven genes leading to the coexpression difference. The differential coexpression analysis (DCEA) method was applied, and 2,856 differentially coexpressed genes (DCGs) were identified. Compared to the differential expression analysis (DEA), it was found that the overlapping of DCGs with the 234 DEGs (*t*-test *p* value less than 0.01) was significant (hypergeometric test *p* = 1.07*e* − 07), indicating that differential expression and differential coexpression are somewhat related to each other, which is consistent with a previous report [17]. However, there are many* Salmonella* infection-related genes identified by the DCEA method. The top one DCG identified is* WASF1*, which is an important gene in the* Salmonella* infection pathway and was not identified as a DEG (expression fold change: 1.08;* t*-test *p* value of 0.34). The protein encoded by* WASF1*, a member of the Wiskott-Aldrich syndrome protein family, plays a critical role downstream of* Rac*, which is a Rho family of small GTPases, in regulating the actin cytoskeleton required for membrane ruffling. This gene associates with an actin nucleation core Arp2/3 complex while enhancing actin polymerization in vitro [32]. Another gene,* CDC42* (fold change = 1.02; *p* = 0.6), a member of the Rho subfamily of actin-organizing small GTP-binding proteins, interacts with* WASF1* and is essential for *S*. Typhimurium entry into host cells [33, 34].* CDC42* was not selected as DCG with a false discovery rate (FDR) of 1.55*E* − 3, but it interacts with genes* PAK7* (fold change = 1.95; *p* = 0.64) [35, 36]*, CDC42EP3* (fold change = 1.05; *p* = 0.81) [37, 38]*, PAK1* (fold change = 0.97; *p* = 0.67) [39]*, PARD6B* (fold change = 0.76; *p* = 0.05) [40, 41]*, PARD6A* (fold change = 1.75; *p* = 0.90) [29, 40], and* IQGAP2* (fold change = 1.58; *p* = 0.12) [42], which are all identified as DCGs. Carow and Rottenberg reported that gene* SOCS3,* which was also identified as a DCG (fold change = 1.54; *p* = 0.15), is a major regulator of infection and inflammation and controls immune homeostasis in physiological and pathological conditions such as infection and autoimmunity [43].* SOCS3 *is a hub gene in the module lightgreen associated with the Jak-STAT signaling pathway, an important pathway for* Salmonella* infection [44]. It is well known that the Jak-STAT pathway can regulate cell growth, apoptosis, immunity, and inflammatory responses and because of its significance in the immune response, the Jak-STAT pathway is often exploited by pathogens [45]. In our study, we found that the Jak-STAT pathway genes were significantly enriched in the module lightgreen which is not detectable in controls. So we think that* SOCS3 *and the other Jak-STAT pathway genes may together regulate the activity of the organism in infection, which leads this module to be differentially coexpressed.

**Figure 6:**
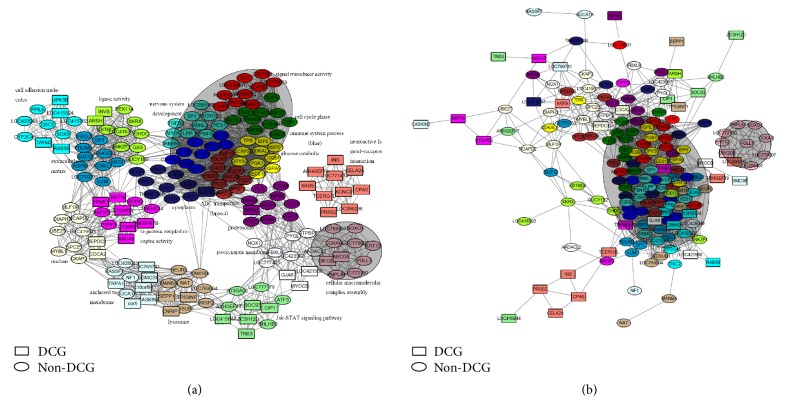
(a) Hub genes' network in the infected group. The node colors represent the module colors: the nodes with shape of rectangle are DCGs and the elliptical nodes are non-DCGs. (b) Hub genes' network in the control group. The node colors represent the module colors: the nodes with shape of rectangle are DCGs and the elliptical nodes are non-DCGs.

The above results showed some specific subnetworks for infection, in spite of a common network existing whether in the control or infected group. We constructed two coexpression networks from the top ten hub genes' expression profiles for each module from the two different conditions. As shown in Figures 6(a) and 6(b), common core networks, including the most preserved modules between infected and control groups, were present. However, some closely interacted subnetworks seen during infection disappeared in the control. These infection-specific subnetworks included genes that are members of the Jak-STAT signaling pathway (module lightgreen); others associated with membrane anchoring (module lightcyan), neuroactive ligand-receptor interaction (module salmon), and lysosomal processing (module tan), which suggested that these subnetworks dysregulated the systems during infection. Although only one dataset was used here, due to the lack of published related microarray datasets, these present results advance our understanding of the cell biology and immunoregulatory pathways involved in* Salmonella *infection in the chicken host.

## Figures and Tables

**Table 1:** Module preservation and functions.

Module	Size	*Z* summary	Function
*Lightyellow*	126	5.42	Nucleus (8.40*E* − 4)

*Lightgreen*	145	0.67	Jak-STAT signaling pathway (9.73*E* − 3)

*Grey60*	145	7.49	Extracellular matrix (5.10*E* − 4)
			Cytoskeleton (7.84*E* − 3)

*Lightcyan*	155	0.52	Anchored to membrane (4.19*E* − 3)

*Midnightblue*	159	5.78	Cytoplasm (1.75*E* − 4);
			Organelle membrane (4.67*E* − 3)
			Endomembrane system (9.27*E* − 3)

*Cyan*	181	8.71	Cell adhesion molecules (3.24*E* − 5)
			Cell adhesion (2.75*E* − 3)

*Salmon*	248	2.14	Neuroactive ligand-receptor interaction (2.60*E* − 6)

*Tan*	287	4.86	Lysosome (4.80*E* − 3)

*Greenyellow*	293	6.58	Ligase activity (3.41*E* − 3)

*Purple*	298	10.93	Proteasome complex (6.56*E* − 6)
			Regulation of cytokine biosynthetic process (3.83*E* − 3)
			Toll-like receptor signaling pathway (9.52*E* − 3)

*Magenta*	357	1.82	G-Protein coupled receptor activity (2.88*E* − 4)

*Pink*	418	13.36	Cellular macromolecular complex assembly (1.00*E* − 03)

*Black*	599	1.97	Postsynaptic membrane (2.35*E* − 3)
			Synapse (2.72*E* − 3)

*Red*	648	14.89	Signal transducer activity (2.07*E* − 4)
			Multicellular organism development (2.70*E* − 4)

*Green*	1056	27.63	Cell cycle phase (9.63*E* − 13)
			DNA replication (3.67*E* − 7)
			Response to DNA damage stimulus (1.45*E* − 6)
			DNA repair (4.11*e* − 6)
			Cytoskeleton organization (3.77*E* − 4)

*Yellow*	1122	32.83	Glucose catabolic process (4.03*E* − 5)
			Glycolysis/gluconeogenesis (1.49*E* − 4)
			Glycolysis (1.79*E* − 4)
			Glucose metabolic process (3.95*E* − 4)

*Brown*	1349	18.99	ABC transporters (1.00*E* − 03)

*Blue*	2581	43.01	Immune system process (1.31*E* − 4)
			Induction of apoptosis (1.98*E* − 4)
			Antigen processing and presentation (2.06*E* − 4)
			Lysosome (3.08*E* − 4)
			Defense response to bacterium (6.06*E* − 3)

*Turquoise*	2998	51.56	Nervous system development (2.20*E* − 15)
			Focal adhesion (2.04*E* − 9)
			Wnt signaling pathway (7.56*E* − 9)
			Regulation of actin cytoskeleton (1.64*E* − 7)
			TGF-beta signaling pathway (4.42*E* − 7)

*Note*. The column “Size” gives the gene numbers contained in every module. “*Z* summary” gives the *z* score of module preservation. “Function” gives the module functions enriched by DAVID.

**Table 2:** Differentially coexpressed modules enriched with differentially coexpressed genes (DCGs).

Module	Size	GSCA.*p*	Hub and DCGs
*Black*	599	0	GJA8, LOC421988, MYOCD
*Magenta*	357	0	C10orf83, PPME1, NRTN, TMEM167B, ABCA5, C10orf58
*Salmon*	248	0	CPA5, PRSS2, ARHGEF19, INS, CELA2A, TCERG1L, LOC396296,
			LOC771434, KCNC2, XKR9
*Lightcyan*	155	5.55*E* − 16	LOC769741, TRPA1, C10orf96, CASKIN2, GMCSF, cor6
*Brown*	1349	2.11*E* − 09	—
*Cyan*	181	1.77*E* − 08	RAB36, LOC415324, UPK3B, CWH43
*Lightgreen*	145	4.05*E* − 07	CIP1, ZC3H12D, TNS3, SOCS3, LOC415844
*Pink*	418	4.20*E* − 07	ABCG8
*Lightyellow*	126	2.10*E* − 05	DEPDC1, CDCA2
*Tan*	287	2.23*E* − 04	TP53INP1, CNRIP1, NEURL, SEPP1
*Midnightblue*	159	1.16*E* − 02	LOC416257
*Greenyellow*	293	2.04*E* − 01	INVS, ARSH
*Grey60*	145	2.10*E* − 01	—
*Purple*	298	2.51*E* − 01	WDR5, NIPA2
*Red*	648	8.66*E* − 01	—
*Blue*	2581	1	—
*Green*	1056	1	—
*Turquoise*	2998	1	—
*Yellow*	1122	1	—

*Note*. The column “Size” gives the gene numbers contained in every module. “GSCA.*p*” gives the *p* value calculated by GSCA for modules between the two different conditions and the *p* value less than 0.05 indicates that the module is significantly differentially coexpressed. “Hub and DCGs” shows the genes which are DCGs in the top ten hub genes.

## Abbreviations

DAVID: Database for Annotation, Visualization, and Integrated Discovery
DCEA: Differential coexpression analysis
DCG: Differentially coexpressed gene
DCL: Differentially coexpressed link
DCM: Differentially coexpressed module
DEA: Differential expression analysis
ECM: Extracellular matrix
FDR: False discovery rate
GO: Gene Ontology
GSCA: Gene-set coexpression analysis
TOM: Topological overlap measure
WGCNA: Weighted gene coexpression network analysis.
